# Development and validation of a nomogram to identify suitable candidates for surgery in malignant small bowel obstruction

**DOI:** 10.1186/s12893-025-03418-2

**Published:** 2025-12-16

**Authors:** Hairui Liu, HaoYang Li, Hao Lu, ZhenLu Li, WenQiang Luo, HongHao Peng, ShiKuan Li

**Affiliations:** 1 Department of Emergency General Surgery, The Affiliated Hospital of Qingdao University, Qingdao, Shandong 266000 China; 2 Department of Emergency General Surgery, Jining No.1 People’s Hospital, Jining, Shandong China

**Keywords:** Malignant small bowel obstruction, Surgery, Palliative, Nomogram

## Abstract

**Background:**

Malignant small bowel obstruction (MSBO) is a severe complication frequently associated with advanced intra-abdominal malignancies, substantially compromising patient survival and quality of life. Surgical management of MSBO remains controversial due to its high postoperative morbidity and mortality. This study aimed to develop and validate a nomogram to identify patients with MSBO who are most likely to benefit from surgical intervention.

**Methods:**

This retrospective study included patients diagnosed with MSBO who underwent surgery at the Affiliated Hospital of Qingdao University between January 2019 and December 2022. Univariate and multivariate analyses were performed, and least absolute shrinkage and selection operator (LASSO) regression was applied in R to identify independent predictors and construct the nomogram. Model performance was evaluated using receiver operating characteristic (ROC) curves, calibration plots, and decision curve analysis (DCA).

**Results:**

A total of 132 patients were included, among whom 69 were in the surgical benefit group (SB group) and 63 in the surgical non-benefit group (SNB group). Serum albumin (mean: 35.67 ± 3.581 vs. 32.45 ± 4.708, *P* = 0.004), maximum small bowel dilation diameter (mean: 0.71 ± 0.076 vs. 0.76 ± 0.085, *P* = 0.047), the ratio of anteroposterior to transverse abdominal diameter (mean: 0.72 ± 0.058 vs. 0.77 ± 0.059, *P* = 0.017), liver metastases (0.20vs 0.56, *P* < 0.01), ascites (0.61vs0.30, *P* < 0.01) were selected as the predictive variables of the nomogram. Through internal validation, we found that the model has good accuracy. Furthermore, the calibration curve indicated the model’s ability to accurately assess individuals who would benefit from surgical intervention, and the Decision Curve Analysis(DCA)curve confirming its potential good clinical utility.

**Conclusion:**

The proposed nomogram, integrating serum albumin, maximum small bowel dilation diameter, the ratio of anteroposterior to transverse abdominal diameter, liver metastases, and ascites, demonstrated robust discriminatory performance and clinical applicability in predicting which patients with malignant small bowel obstruction (MSBO) are likely to benefit from surgery. This tool may assist clinicians in making individualized, evidence-based treatment decisions. Prospective multicenter validation is needed to confirm its utility and facilitate its incorporation into routine clinical practice.

**Supplementary information:**

The online version contains supplementary material available at 10.1186/s12893-025-03418-2.

## Introduction

Malignant bowel obstruction (MBO) is defined as mechanical blockage of the bowel caused by primary or metastatic malignancies. According to the Clinical Protocol Committee, the diagnostic criteria for MBO include: (1) clinical evidence of bowel obstruction based on history, physical examination, or radiographic findings, (2) obstruction occurring beyond the ligament of Treitz, (3) primary intra-abdominal cancer with incurable disease, and (4) primary non-intra-abdominal cancer with evident intraperitoneal disease [1]. The reported incidence of MBO in advanced malignancies ranges from 3% to 15% [2], and among these cases, approximately 10–28% occur in patients with digestive malignancies, whereas about 51% are seen in those with gynecologic cancers [3]. Occasionally, MBO may occur in advanced breast cancer, melanoma, or other malignancies.

Patients are typically diagnosed with MBO at an advanced stage, often experiencing profound deterioration in quality of life and significant economic burden, and till now, the choice between surgical and non-surgical treatments for this patient group remains controversial. Jon C. Henry et al. reported that surgery was associated with improved survival, with a median survival of 6.6 months in surgical patients versus 1.7 months in non-surgical patients [4]. In contrast, Sarah Bateni et al. analyzed data from 4,576 patients and found no significant difference in the 30-day versus 90-day survival rates between the surgical and non-surgical groups [5]. Similarly, Tseng demonstrated that surgical intervention for disseminated malignant tumors is associated with high postoperative mortality rates [6].

MBO can be classified as malignant small bowel obstruction (MSBO) or malignant large bowel obstruction (MLBO) depending on the site of obstruction. MLBO is often caused by a single, clearly localized lesion—most commonly a primary colorectal tumor—making surgical or interventional management relatively straightforward [7]. By contrast, MSBO is frequently related to diffuse peritoneal carcinomatosis with multiple levels of obstruction, which greatly limits surgical options and complicates decision-making. Given these differences, MSBO represents a particularly challenging entity that warrants specific prognostic tools [8].

Given these distinctions, MSBO presents unique diagnostic and therapeutic challenges. This study aims to develop a predictive model to assist in identifying MSBO patients who are most likely to benefit from surgical intervention, thereby supporting more precise and individualized treatment decision-making.

## Methods

The study protocol was approved by the Ethics Committee of the Affiliated Hospital of Qingdao University (QYFY WZLL 28779).

Clinical data were retrospectively retrieved from the hospital information system (HIS) for patients diagnosed with MSBO who underwent surgery at the Affiliated Hospital of Qingdao University between January 2019 and December 2022.

During the study period (2019–2022), our institution maintained emergency surgery for bowel obstruction as a priority service despite the COVID-19 pandemic. We reviewed the medical records for documented SARS-CoV-2 infection and COVID-19-related complications; only a small number of patients had a history of COVID-19, and no deaths in this cohort were directly attributed to COVID-19.

### Inclusion criteria were as follows

1. Age ≥ 18 years;
2. Clinical or imaging confirmation of small bowel obstruction distal to the ligament of Treitz;
3. Etiology attributable to a primary or secondary intra-abdominal malignancy;
4. Presence of an intra-abdominal malignancy not amenable to radical resection;
5. Availability of complete medical records.

### Exclusion criteria included

1. Age < 18 years;
2. Concomitant colonic obstruction;
3. Intra-abdominal malignancy amenable to radical resection;
4. Gastrointestinal perforation or bowel necrosis;
5. Incomplete medical records;
6. Loss to follow-up.

### Grouping criteria

**Fig. 1:**
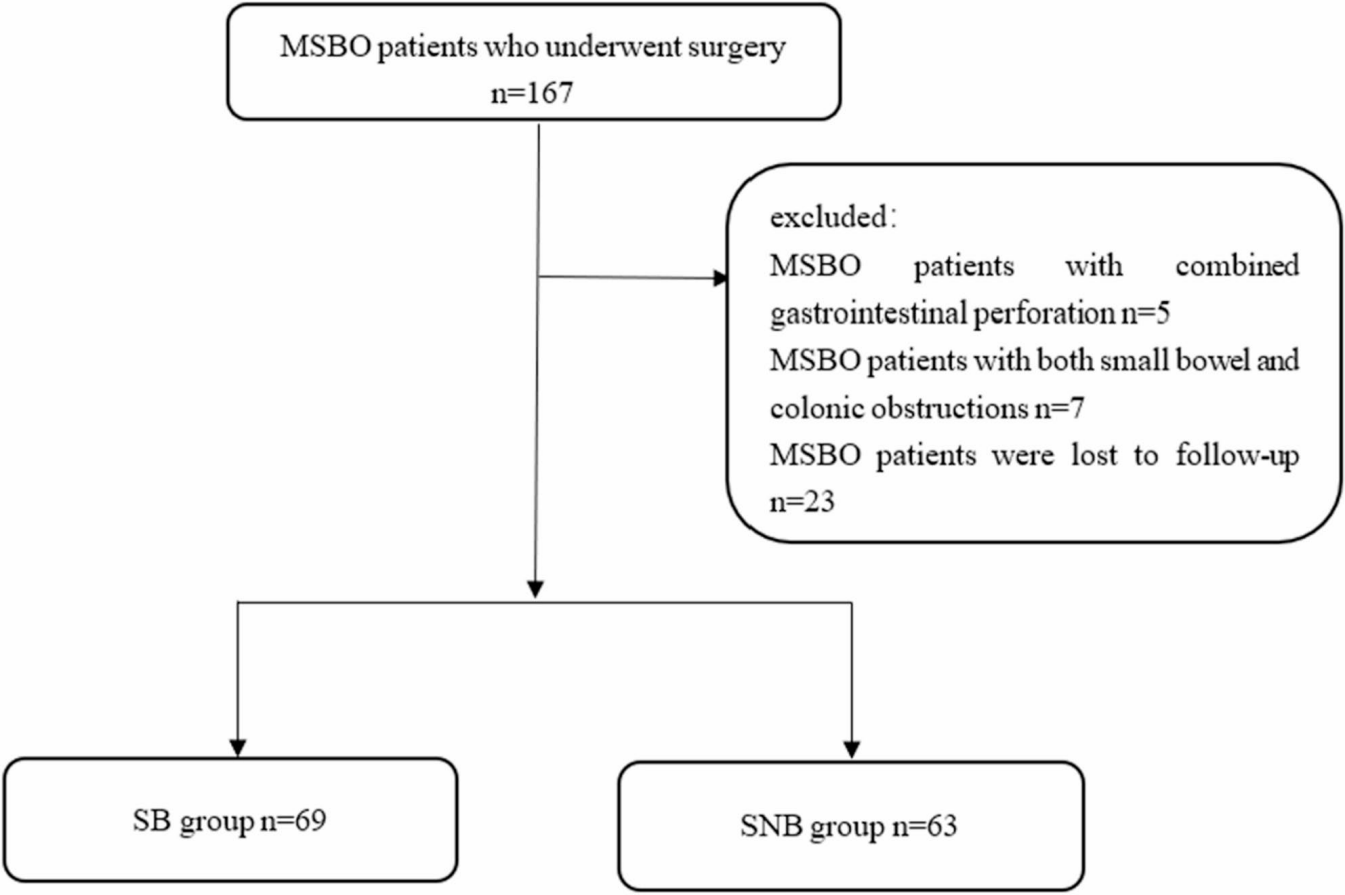
Flowchart for patients

Patients were stratified into a surgical benefit (SB) group and a surgical non-benefit (SNB) group(Fig. 1). The classification was based on postoperative survival, with the SB group comprising patients surviving > 90 days postoperatively, and the SNB group comprising those surviving ≤ 90 days, consistent with prior literature suggesting that survival beyond 90 days correlates with sustained symptom relief and extended overall survival [9–11].

### Data collection

Because this was a retrospective study, clinical signs and symptoms (such as abdominal pain, vomiting, abdominal distension and bowel sounds) were not documented in a standardized manner across all patients. To minimize information bias and ensure reproducibility, we therefore restricted the candidate predictors to objectively measured variables, including laboratory parameters and radiologic findings.

Ascites was assessed on preoperative contrast-enhanced abdominal CT scans by experienced radiologists. The volume of ascites was categorized into three groups: no ascites, small amount of ascites, and large amount of ascites. Small-volume ascites was defined as a thin fluid layer limited to the hepatorenal, perisplenic or pelvic recesses without causing bowel flotation, whereas large-volume ascites was defined as diffuse intra-abdominal fluid with centrally floating bowel loops and/or a maximal fluid depth ≥ 3 cm in at least two abdominal quadrants.

Baseline demographic and clinical variables included age, sex, length of hospital stay (LHS), duration of bowel obstruction (DBO), and history of tumor-related surgery (HTRS). Laboratory parameters included serum albumin (Alb), C-reactive protein (CRP), hemoglobin (Hb), potassium (K), white blood cell count (WBC), and neutrophil count (NEU).

**Fig. 2:**
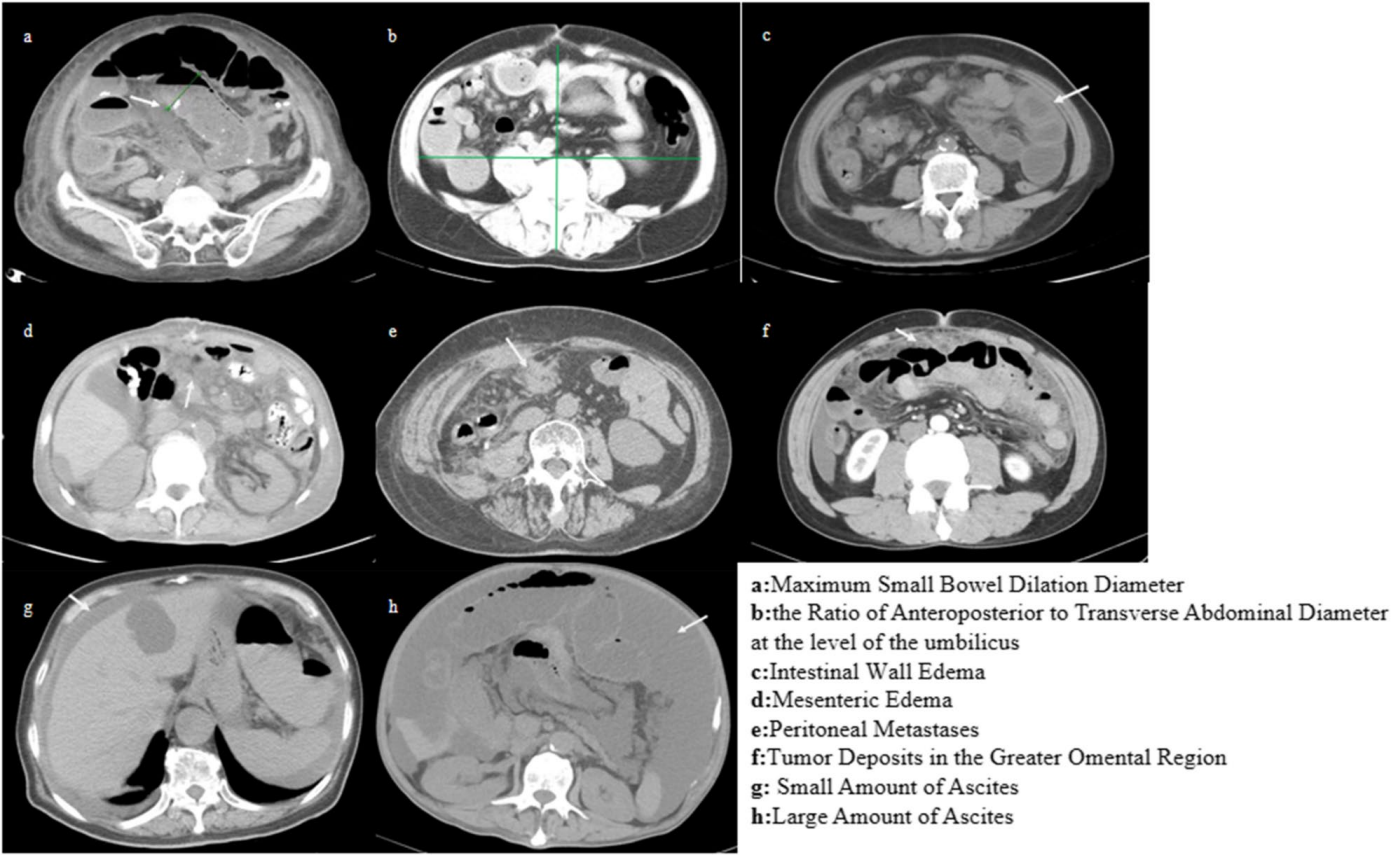
Imaging features

Radiologic variables assessed on preoperative imaging included: maximum small bowel dilation diameter (MSBDD), ratio of anteroposterior to transverse abdominal diameter (RATAD), intestinal wall edema (IWE), mesenteric edema (ME), peritoneal metastases (PM), tumor deposits in the greater omental region(TDGOR), liver metastases (LM), and presence/volume of ascites (Fig. 2).

### Statistical analysis

Statistical analysis was performed using the SPSS v27.0 software. Measurement data were analyzed with either the independent sample t-test or the Kruskal-Wallis test, while count data were assessed using the chi-square test or Fisher’s exact probability method. A significance level of *P* < 0.05 was used to indicate statistical significance. In addition, we utilized the R code to perform lasso regression for predictor selection and to construct the nomogram model. Subsequently, the receiver operating characteristic (ROC) curve was used to calculate the area under the ROC curve. Internal validation was performed through Bootstrap resampling, and a calibration curve was plotted to evaluate the model’s accuracy for risk estimation. Furthermore, we employed decision curve analysis (DCA) to assess the clinical utility of the predictive model and determine its alignment with clinical requirements.

All variables with a P value < 0.10 in univariate analyses, as well as age and sex based on clinical relevance, were included as candidate predictors in the LASSO regression. This approach was chosen to balance model parsimony with the risk of omitting potentially important predictors in the context of a limited sample size. The LASSO procedure identified five variables with non-zero coefficients—serum albumin, MSBDD, RATAD, liver metastases and ascites—which were subsequently used to construct the nomogram.

## Results

A total of 167 patients diagnosed with MSBO were initially identified using the HIS system. After excluding 5 patients with combined gastrointestinal perforation and 7 patients with both small bowel and colonic obstructions, and considering the loss to follow-up of 23 patients.A final cohort of 132 patients was selected for analysis.

### Baseline characteristics

Univariate analysis results for both groups are presented in Table 1. No significant differences were observed between the SB and SNB groups regarding age, sex, LHS, or DBO.

**Table 1:** Univariate analysis of variables in the predictive model

	SB-group (*n* = 69)	SNB-group (*n* = 63)	Difference value	95%CI	*P* value
Age	62.26 ± 12.245	59.27 ± 14.036	1.02	0.982–1.055	0.335
Sex			1.29	0.481–3.435	0.449
Male	46(0.67)	38(0.60)			
Female	23(0.33)	25(0.40)			
Alb(g/L)	35.67 ± 3.581	32.45 ± 4.708	1.21	1.065–1.376	0.004
Hb(g/L)	115.64 ± 19.850	108.88 ± 21.014	1.02	0.993–1.04	0.171
K(mmol/L)	4.06 ± 0.474	4.23 ± 0.483	0.47	0.169–1.302	0.146
MSBDD	44.71 ± 11.695	39.22 ± 11.278	1.04	1–1.089.089	0.047
RATAD	0.71 ± 0.076	0.76 ± 0.085	0.00	0–0.157.157	0.012
LHS (d)	13(9–21)	12(8.5–16.5)	1.01	0.968–1.063	0.554
DBO (d)	10(5–25)	14(7–27.5.5)	0.99	0.964–1.022	0.62
CRP (mg/L)	5.36(3.17–22.92)	11.63(4.49–46.76)	1.00	0.996–1.004	0.926
WBC (10^9^/L)	5.38(4.69–6.66)	6.19(3.69,8.12)	0.87	0.702–1.078	0.204
NEU (10^9^/L)	3.36(2.73–4.66)	4.11(2.81–5.53)	0.78	0.601–1.001	0.040
			OR		
HTRS	60(0.87)	51(0.81)	2.55	0.759–8.566	0.346
IWE	35(0.51)	23(0.64)	1.35	0.523–3.499	0.100
ME	34(0.49)	45(0.62)	0.36	0.132–0.959	0.010
PM	58(0.84)	57(0.90)	0.68	0.15–3.088	0.272
TDGOR	37(0.54)	43(0.68)	0.37	0.135–1.03	0.086
LM	14(0.20)	35(0.56)	0.33	0.123–0.87	< 0.01
Ascites			0.33	0.115–0.916	< 0.01
No	42(0.61)	19(0.30)			
Small amount	24(0.35)	30(0.48)			
Large amount	3(0.04)	14(0.22)			

*SB-group *Surgery benefit group, *SNB-group *Surgery non-benefit group, *MSBDD *Maximum small bowel dilation diameter, *RATAD *The ratio of anteroposterior to transverse abdominal diameter, *LHS *Length of hospital stay, *DBO *Duration of bowel obstruction, *CRP *C-reactive protein, *WBC *White blood cell, *NEU *Neutrophil count, *HTRS *History of tumor-related surgery, *IWE *Intestinal wall edema, *ME *Mesenteric edema, *PM *Peritoneal metastases, *TDGOR *Tumor deposits in the greater omental region, *LM *Liver metastases

### Laboratory finding

Compared with the SNB group, the SB group had significantly higher serum albumin levels (35.67 ± 3.581 vs. 32.45 ± 4.708 g/L, *P* = 0.004) and lower neutrophil counts (3.36 [2.73–4.66] vs. 4.11 [2.81–5.53] × 10⁹/L, *P* = 0.040). No significant intergroup differences were observed for Hb, K, CRP, or WBC.

### Radiologic findings

The SB group exhibited greater small bowel dilation (MSBDD: 44.71 ± 11.695 vs. 39.22 ± 11.278 mm, *P* = 0.047) and a lower RATAD (0.71 ± 0.076 vs. 0.76 ± 0.085, *P* = 0.012). The SNB group had a higher prevalence of mesenteric edema (0.49 vs. 0.62, *P* = 0.010), liver metastases (0.20 vs. 0.56, *P* < 0.01), and ascites (0.61 vs. 0.30, *P* < 0.01). There were no statistically significant differences in the prevalence of HTRS, PM, or TDGOR between groups.

On univariate analysis, higher serum albumin levels, greater maximum small bowel dilation diameter, lower anteroposterior-to-transverse abdominal diameter ratio, lower neutrophil count, absence of mesenteric edema, absence of liver metastases and lower volume of ascites were significantly associated with surgical benefit (*P* < 0.05 for all; Table 1). These variables were therefore considered as candidates for multivariable modelling and subsequent LASSO regression.

### Multivariate analysis

Variables with *P* < 0.05 in univariate analysis were included in the multivariate logistic regression model (Table 2). Liver metastases (OR 3.61, 95% CI: 1.05–12.36, *P* = 0.041) and ascites (OR 11.06, 95% CI: 0.99–123.83, *P* = 0.043) were identified as independent risk factors for poor surgical benefit.

**Table 2:** Multivariate analysis of variables in the predictive model

	OR	95%CI	*P*
Alb	0.89	0.77–1.03	0.109
RATAD	224.24	0.12–430234.23.12.23	0.16
ME	2.24	0.59–8.45	0.235
LM	3.61	1.05–12.36	0.041
Ascites	11.06	0.99–123.83.99.83	0.043

### Nomogram construction and validation

**Fig. 3:**
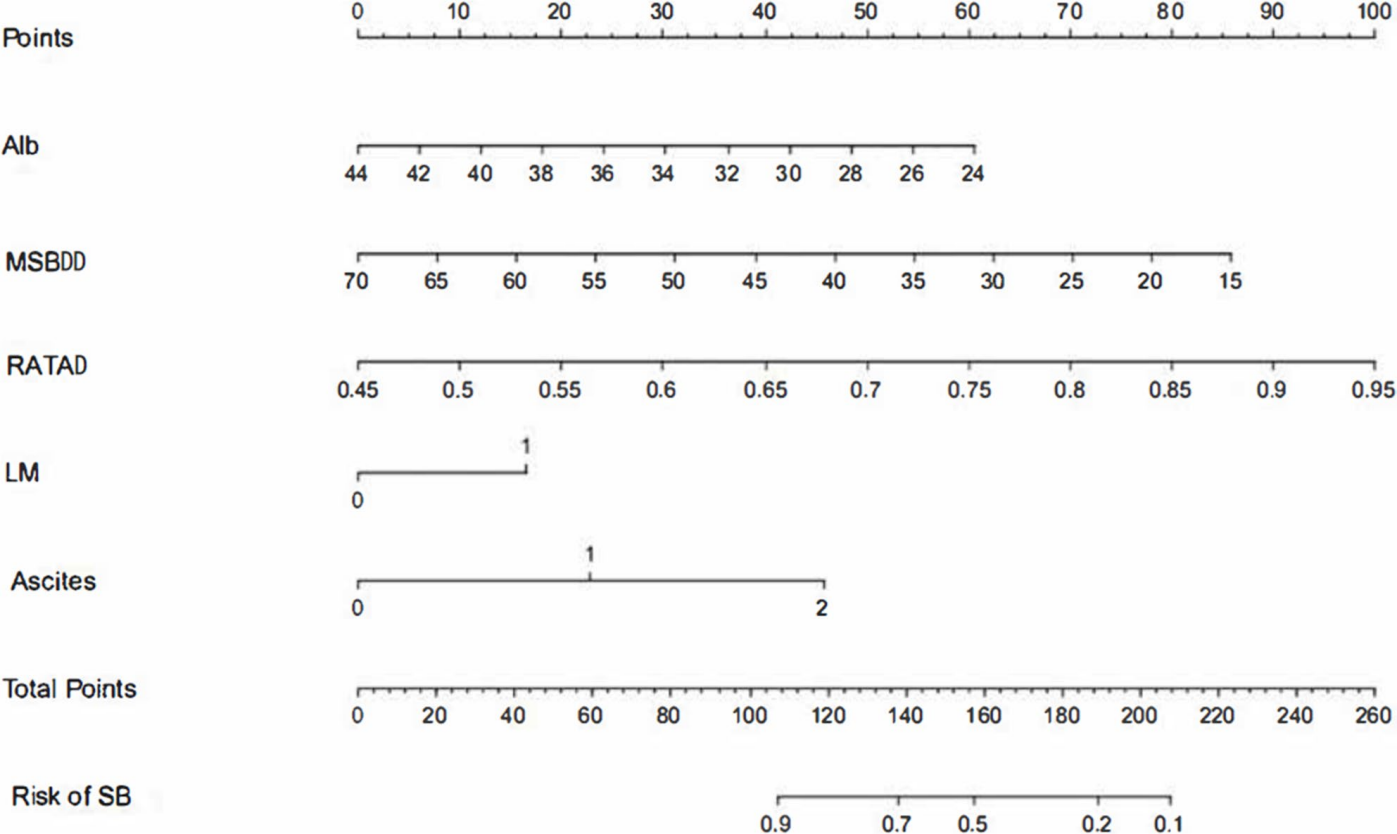
Predictive models for risk of surgical benefit composed of Alb, MSBDD, RATAD, LM and Ascites

**Fig. 4:**
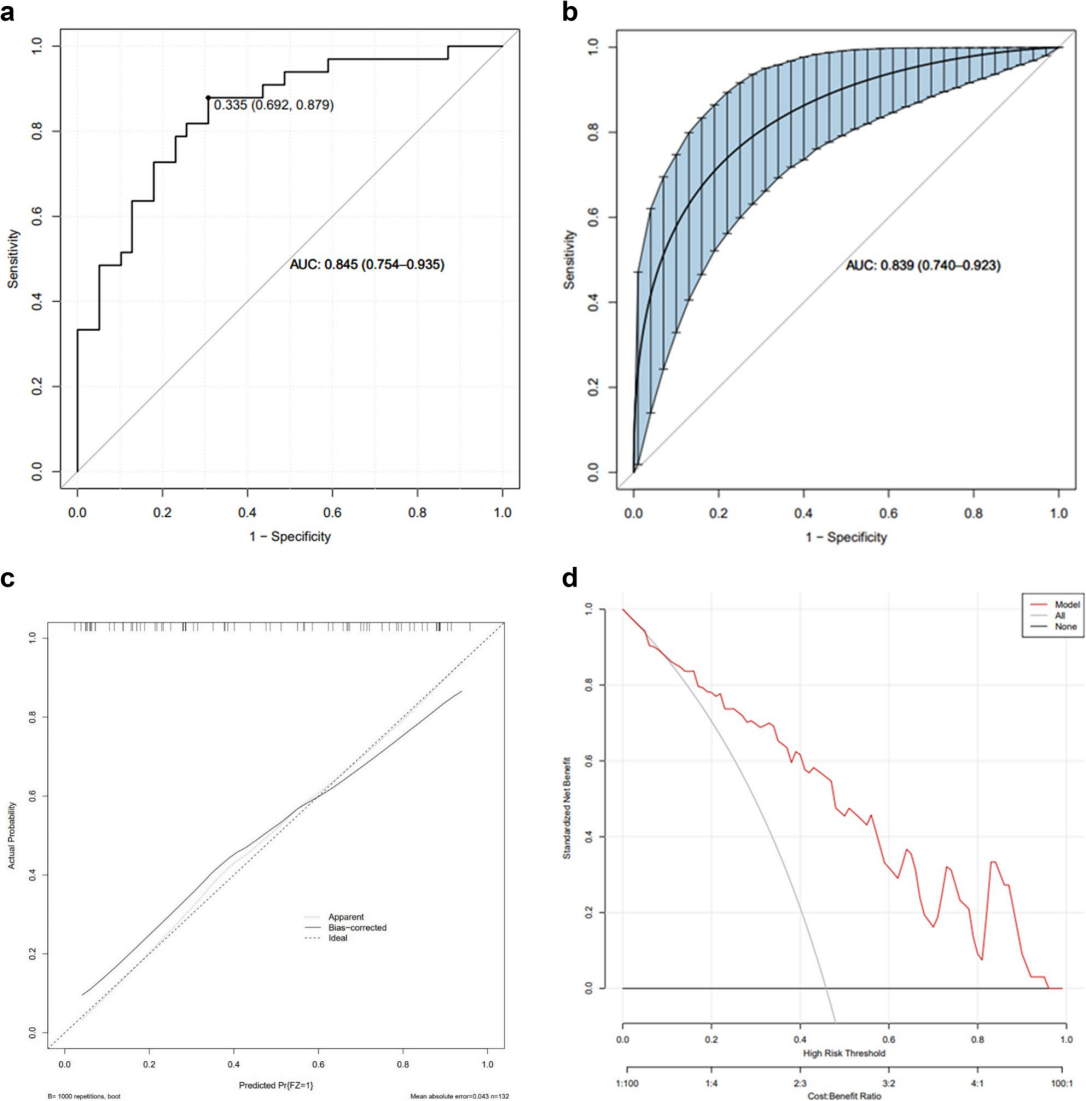
ROC curve and AUC of the prediction model. **a** ROC curve and AUC of the prediction model. **b** Bootstrap (internal validation). **c** Calibration curves for predictive models. **d** Clinical decisions curves analysis for predictive modeling

LASSO regression identified five predictive variables for the final nomogram: Alb, MSBDD, RATAD, LM, and ascites (Fig. 3). The model demonstrated good discriminatory performance, with an AUC of 0.845 (95% CI: 0.754–0.935) (Fig. 4a). Using a cut-off value of 0.335, the model achieved a sensitivity of 0.879 and specificity of 0.692, corresponding to the highest Youden index.

Internal validation via bootstrap resampling yielded a mean absolute error of 0.043 (< 0.05), indicating excellent calibration (Fig. 4b and c). DCA showed that the net clinical benefit of the model was superior to either the “treat all” or “treat none” strategies across a wide range of threshold probabilities (Fig. 4d), suggesting strong potential for clinical application.

## Discussion

Many patients with MSBO, together with their families, frequently choose non-surgical management, a decision often shaped by the emotional toll of prolonged cancer treatment and the perception that the disease has entered its terminal stage. Surgeons’ therapeutic choices are likewise constrained by multiple considerations, including the high rates of postoperative complications, prolonged hospitalization, and increased mortality observed in this population [12]. In the present study, we retrospectively reviewed data from 132 surgically managed MSBO patients and, for the first time, dichotomized postoperative survival into two outcome categories. This approach enabled the development of a predictive model intended to support surgeons in making more precise and individualized treatment decisions.

Certain clinicopathological factors, such as primary tumor origin, metastatic pattern, and previous abdominal or obstruction-related surgery, were not uniformly documented across all patients and therefore could not be included in our analysis. This represents a limitation of our retrospective design, and future prospective studies with standardized data collection are required.

In our analysis, ascites emerged as an independent prognostic factor for adverse surgical outcomes in MSBO, consistent with the observations of Wancata, Henry, and Tseng [4, 6, 13]. The surgical risk appears to increase proportionally with ascites volume. Malignant ascites is widely recognized as a poor prognostic marker, with median survival averaging approximately 20 weeks after diagnosis [14], This unfavorable prognosis is likely related to tumor-derived vascular endothelial growth factor (VEGF) and associated mediators that increase vascular permeability [15–17]. Given that the majority of MSBO patients present with peritoneal metastases, routine intraoperative cytologic evaluation of ascitic fluid may be unnecessary, as cytology is positive in nearly 97% of peritoneal carcinomatosis cases [14]. Palliative surgery in this setting serves dual purposes: reducing tumor burden and restoring bowel continuity, while simultaneously mitigating ascites formation through intraoperative hyperthermic intraperitoneal perfusion. In a retrospective series by Valle, 94% of 52 patients with malignant ascites experienced complete resolution after one month of intraperitoneal thermoperfusion [18]. Similarly, Randle’s statistical analysis demonstrated that cytoreductive surgery combined with hyperthermic intraperitoneal chemotherapy achieved control of malignant ascites in approximately 93% of cases [19].

Our findings also identified liver metastases as an independent prognostic risk factor for surgical outcomes in MSBO. This aligns with prior evidence showing that hepatic metastases are strongly linked to poor prognosis in intra-abdominal malignancies, including peritoneal, colorectal, and ovarian cancers [20–23]. The liver’s predisposition as the predominant site of distant metastasis in such cancers is thought to arise from its distinct microenvironment and favorable hemodynamic conditions, which facilitate tumor cell adhesion and growth [24, 25]. In MSBO patients, surgical management of hepatic metastases is rarely feasible because intra-abdominal disease is typically unresectable (aside from the hepatic lesions) and patients often present with compromised overall health. Moreover, surgical intervention in this context carries a substantial risk of postoperative complications. Once bowel obstruction is relieved and the patient’s general condition improves, non-surgical modalities—including radiotherapy, chemotherapy, immunotherapy, targeted agents, and interventional therapies—may be more appropriate.

Serum albumin remains a key biomarker for evaluating nutritional status, and numerous studies have linked hypoalbuminemia to poor prognosis in malignant tumors. Henry et al. reported that low albumin levels were associated with a significantly increased risk of 30-day postoperative mortality in MSBO patients [4, 26]. Albumin status is also closely tied to perioperative outcomes; for instance, Issangya et al. demonstrated that decreased perioperative albumin levels can adversely affect results in major abdominal surgery [27]. In MSBO, prolonged tumor burden and impaired oral intake frequently lead to negative nitrogen balance—where protein catabolism exceeds synthesis—resulting in reduced albumin concentrations. Addressing malnutrition is therefore essential, and the use of total parenteral nutrition (TPN) has been shown to prolong survival and improve quality of life in this patient population [28, 29]. Nonetheless, the true impact of preoperative TPN or exogenous human albumin supplementation on albumin levels, overall condition, and surgical risk remains uncertain, as high-quality clinical trials are lacking.

Intraoperatively, most MSBO patients demonstrated disseminated tumor deposits adherent to the intestinal and peritoneal surfaces. Tumor implantation on the peritoneum can trigger peritoneal fibrosis, initiated by the rapid transformation of adjacent mesenchymal cells into fibroblasts in response to tumor cell invasion. This fibrotic process typically originates in the pelvic peritoneum, diaphragmatic peritoneum, and the greater and lesser omenta, and may subsequently extend to the small bowel mesentery—potentially leading to mesenteric contracture and, in some cases, the development of a “frozen pelvis” [30, 31]. While our prediction model assigns lower scores with increasing small bowel lumen dilation, it is important to emphasize that the choice of surgical technique depends heavily on the pattern and extent of tumor spread, and this choice can substantially influence outcomes. Surgical strategies may include tumor debulking, stoma formation, bypass procedures, or combinations thereof. However, given the limited sample size and the resulting statistical constraints, we did not incorporate surgical approach as a variable in the current analysis.

Persistent bowel obstruction, progressive ascites, and peritoneal fibrosis can act synergistically to elevate intra-abdominal pressure, potentially culminating in abdominal compartment syndrome. Such pressure elevation further aggravates the clinical course in patients with MSBO. The Kron technique, as modified by the World Society of the Abdominal Compartment Syndrome, remains the most widely adopted clinical method for intra-abdominal pressure measurement [32]. In this retrospective study, however, intra-abdominal pressure data were limited. Notably, Bouveresse et al. demonstrated a strong correlation between the ratio of anteroposterior to transverse abdominal diameters and directly measured intra-abdominal pressure, suggesting this ratio as a reliable surrogate marker [33]. Accordingly, we incorporated CT-derived measurements of the anteroposterior-to-transverse diameter ratio into our model, thereby enhancing its practicality while maintaining objectivity in estimating intra-abdominal pressure.

A recent Korean study reported a marked survival advantage for patients receiving palliative postoperative chemotherapy compared with those undergoing palliative surgery alone. Median survival was 3.5 months for the surgery-only group versus 12.3 months for the surgery-plus-chemotherapy group (*p* < 0.001) [34]. These data suggest that a multimodal approach—combining palliative surgery to relieve obstruction with subsequent adjuvant therapy—may be more effective in improving quality of life and prolonging survival in appropriately selected MSBO patients.

Patients with MSBO often endure substantial physical and psychological distress over prolonged periods, and their treatment should not be disregarded solely due to the presence of advanced malignancy. The primary therapeutic objectives in MSBO remain the improvement of quality of life and the extension of survival. In the future, individualized, multidisciplinary treatment strategies will likely play an increasingly pivotal role in optimizing outcomes for this challenging condition.

***6.Limitations***: This study was a single-center retrospective study with a relatively small sample size, which was unable to externally validate the model, which may have an impact on the results and accuracy of the model. Differences in surgeons and surgical methods may affect surgical outcomes. Additionally, some patients did not have complete preoperative examinations, which prevented us from including other indicators (such as tumor marker detection and procalcitonin). Initially, we attempted to subdivide the groups based on the different surgical methods, but due to the small sample size in each group, there was a significant bias. We did not record the details of the adjuvant therapy and duration for patients after surgery, which will affect the overall survival results. Due to the patients being in the late stage of cancer, the follow-up was relatively limited, and most patients passed away during the follow-up period. Some family members refused to provide further details. Fourth, although emergency surgery for bowel obstruction remained available throughout the COVID-19 pandemic at our institution, we cannot fully exclude indirect effects of the pandemic on perioperative management, resource allocation or long-term follow-up, which may have influenced patient outcomes.

In the present study, we dichotomized postoperative survival at 90 days to define ‘surgical benefit’, in line with previous literature suggesting that survival beyond this time point is associated with sustained symptom relief and clinically meaningful prolongation of life. However, this approach inevitably simplifies the complex heterogeneity among patients with MSBO. Differences in performance status, comorbidities, tumor biology and patient preferences are not fully captured by a single time-based cut-off. Our nomogram should therefore be interpreted as an adjunct to, rather than a replacement for, comprehensive clinical judgement and multidisciplinary discussion.

## Conclusion

This study developed and internally validated a nomogram incorporating serum albumin, maximum small bowel dilation diameter, the ratio of anteroposterior to transverse abdominal diameter, liver metastases, and ascites to predict surgical benefit in patients with malignant small bowel obstruction (MSBO). The model demonstrated high discriminatory accuracy, good calibration, and favorable clinical utility, offering a practical tool to guide individualized surgical decision-making. Our findings underscore the importance of careful preoperative assessment in identifying patients most likely to benefit from surgery, thereby optimizing outcomes while minimizing unnecessary risk. Future multicenter prospective studies with larger cohorts and external validation are warranted to confirm the generalizability of this predictive model and to explore its integration into multidisciplinary treatment algorithms for MSBO.

## Supplementary information

Supplementary Material 1.

## Data Availability

The datasets used and/or analyzed during the current study are under further analysis and are available from the corresponding author on reasonable request.

## Abbreviations

MSBO: Malignant small bowel obstruction
LASSO: Least absolute shrinkage and selection operator
ROC: Receiver operating characteristic
DCA: Decision curve analysis
SB: Surgical benefit
MLBO: Malignant large bowel obstruction
DBO: Duration of bowel obstruction
HTRS: History of tumor-related surgery
ALB: Serum albumin
CRP: C-reactive protein
WBC: White blood cell count
NEU: Neutrophil count
MSBDD: Maximum small bowel dilation diameter
RATAD: Ratio of anteroposterior to transverse abdominal diameter
IWE: Intestinal wall edema
ME: Mesenteric edema
PM: Peritoneal metastases
TDGOR: Tumor deposits in the greater omental region
LM: Liver metastases
TPN: Total parenteral nutrition

## References

[1] Anthony T, Baron T, Mercadante S, Green S, Chi D, Cunningham J, et al. Report of the clinical protocol committee: development of randomized trials for malignant bowel obstruction. J Pain Symptom Manage. 2007;34(1):S49–59. 10.1016/j.jpainsymman.2007.04.011. 17544243 10.1016/j.jpainsymman.2007.04.011

[2] Huang XY, Xue J, Gao M, Qin QY, Ma TH, Li XY, Wang H. Medical management of inoperable malignant bowel obstruction. Ann Pharmacother. 2021;55(9):1134–45. [PMID: WOS:000674723600008 DOI: 10.1177/1060028020979773]. 33345552 10.1177/1060028020979773

[3] Cousins SE, Tempest E, Feuer DJ. Surgery for the resolution of symptoms in malignant bowel obstruction in advanced gynaecological and gastrointestinal cancer. Cochrane Database Syst Rev. 2016;1(3):74. 10.1002/14651858.CD002764.pub2. 10.1002/14651858.CD002764.pub2 PMC7101053 26727399

[4] Henry JC, Pouly S, Sullivan R, Sharif S, Klemanski D, Abdel-Misih S, et al. A scoring system for the prognosis and treatment of malignant bowel obstruction. Surgery. 2012;152(4):747–57. 22929404 10.1016/j.surg.2012.07.009 PMC3792226

[5] Bateni SB, Gingrich AA, Stewart SL, Meyers FJ, Bold RJ, Canter RJ. Hospital utilization and disposition among patients with malignant bowel obstruction: a population-based comparison of surgical to medical management. BMC Cancer. 2018;18:10. 10.1186/s12885-018-5108-9. 30477454 10.1186/s12885-018-5108-9 PMC6258444

[6] Tseng WH, Yang XW, Wang H, Martinez SR, Chen SL, Meyers FJ, et al. Nomogram to predict risk of 30-day morbidity and mortality for patients with disseminated malignancy undergoing surgical intervention. Ann Surg. 2011;254(2):333–8. 10.1097/SLA.0b013e31822513ed. 21677562 10.1097/SLA.0b013e31822513ed

[7] Huang X, Xue J, Gao M, Qin Q, Ma T, Li X, et al. Medical management of inoperable malignant bowel obstruction. Ann Pharmacother. 2021;55(9):1134–45 ([PMID: 33345552 DOI: 10.1177/1060028020979773]). 33345552 10.1177/1060028020979773

[8] Nakagawa K, Ishibe A, Ohya H, Ozawa M, Suwa Y, Watanabe J, et al. Effects of neoadjuvant chemotherapy for patients with obstructive colon cancer: a multicenter propensity score-matched analysis (YCOG2101). Ann Gastroenterol Surg. 2024;8(2):262–72. 10.1002/ags3.12736. 38455492 10.1002/ags3.12736 PMC10914701

[9] Tuca A, Guell E, Martinez-Losada E, Codorniu N. Malignant bowel obstruction in advanced cancer patients: epidemiology, management, and factors influencing spontaneous resolution. Cancer Manag Res. 2012;4:159–69. 22904637 10.2147/CMAR.S29297 PMC3421464

[10] Turrado-Rodriguez V, Serra RT, Besa A, Llerena GC, Viladot M, Sevillano XM. MALIGNANT SMALL BOWEL OBSTRUCTION: OUTCOMES OF PALLIATIVE SURGERY. Br J Surg. 2021. 10.1093/bjs/znab160.015.

[11] Krouse RS, Anderson GL, Arnold KB, Thomson CA, Nfonsam VN, Al-Kasspooles MF, Walker JL, Sun V, Alvarez Secord A, Han ES, Leon-Takahashi AM, Isla-Ortiz D, Rodgers P, Hendren S, Sanchez Salcedo M, Laryea JA, Graybill WS, Flaherty DC, Mogal H, Miner TJ, Pimiento JM, Kitano M, Badgwell B, Whalen G, Lamont JP, Guevara OA, Senthil MS, Dewdney SB, Silberfein E, Wright JD, Friday B, Fahy B, Anantha Sathyanarayana S, O’Rourke M, Bakitas M, Sloan J, Grant M, Deutsch GB, Deneve JL. Surgical versus non-surgical management for patients with malignant bowel obstruction (S1316): a pragmatic comparative effectiveness trial. Lancet Gastroenterol Hepatol. 2023;8(10):908–18. 10.1016/s2468-1253(23)00191-7]. [PMID: 37541263 PMCID: PMC. 37541263 10.1016/S2468-1253(23)00191-7 PMC10530384

[12] Olson TJP, Pinkerton C, Brasel KJ, Schwarze ML. Palliative surgery for malignant bowel obstruction from carcinomatosis A systematic review. JAMA Surg. 2014;149(4):383–92. 10.1001/jamasurg.2013.4059]. [PMID: WOS:000334609900017. 24477929 10.1001/jamasurg.2013.4059 PMC4030748

[13] Wancata LM, Abdelsattar ZM, Suwanabol PA, Campbell DA, Hendren S. Outcomes after surgery for benign and malignant small bowel obstruction. J Gastrointest Surg. 2017;21(2):363–71. 10.1007/s11605-016-3307-8]. [PMID: WOS:000393825300020. 27783343 10.1007/s11605-016-3307-8 PMC5263174

[14] Sangisetty SL, Miner TJ. Malignant ascites: A review of prognostic factors, pathophysiology and therapeutic measures. World J Gastrointest Surg. 2012;4(4):87–95. 10.4240/wjgs.v4.i4.87]. [PMID: MEDLINE:22590662. 22590662 10.4240/wjgs.v4.i4.87 PMC3351493

[15] Senger DR, Galli SJ, Dvorak AM, Perruzzi CA, Harvey VS, Dvorak HF. Tumor cells secrete a vascular permeability factor that promotes accumulation of Ascites fluid. Sci (New York NY). 1983;219(4587):983–5. 10.1126/science.6823562]. [PMID: MEDLINE:6823562. 10.1126/science.6823562 6823562

[16] Garrison RN, Kaelin LD, Galloway RH, Heuser LS. Malignant ascites. Clinical and experimental observations. Ann Surg. 1986;203(6):644–51. 10.1097/00000658-198606000-00009] . [PMID: MEDLINE:3718029. 3718029 10.1097/00000658-198606000-00009 PMC1251196

[17] Nagy JA, Benjamin L, Zeng HY, Dvorak AM, Dvorak HF. Vascular permeability, vascular hyperpermeability and angiogenesis. Angiogenesis. 2008;11(2): 109–19 [PMID: WOS:000256502000001. 10.1007/s10456-008-9099-z.] 10.1007/s10456-008-9099-z PMC2480489 18293091

[18] Valle M, Van der Speeten K, Garofalo A. Laparoscopic hyperthermic intraperitoneal peroperative chemotherapy (HIPEC) in the management of refractory malignant ascites: a multi-institutional retrospective analysis in 52 patients. J Surg Oncol. 2009;100(4):331–4. 10.1002/jso.21321. 19697441 10.1002/jso.21321

[19] Randle RW, Swett KR, Swords DS, Shen P, Stewart JH, Levine EA, et al. Efficacy of cytoreductive surgery with hyperthermic intraperitoneal chemotherapy in the management of malignant ascites. Ann Surg Oncol. 2014;21(5):1474–9. 10.1245/s10434-013-3224-y. 23982251 10.1245/s10434-013-3224-y PMC4090137

[20] Ayantunde AA, Parsons SL. Pattern and prognostic factors in patients with malignant ascites: a retrospective study. Ann Oncol. 2007;18(5):945–9. 10.1093/annonc/mdl499. 17298959 10.1093/annonc/mdl499

[21] Zhang S, Gao F, Luo J, Yang J. Prognostic factors in survival of colorectal cancer patients with synchronous liver metastasis. Colorectal Dis. 2010;12(8):754–61. 10.1111/j.1463-1318.2009.01911.x. 19508508 10.1111/j.1463-1318.2009.01911.x

[22] Tsilimigras DI, Brodt P, Clavien PA, Muschel RJ, D’Angelica MI, Endo I, et al. Liver metastases. Nat Rev Dis Primers. 2021;7(1):23. 10.1038/s41572-021-00261-6. 33859205 10.1038/s41572-021-00261-6

[23] Dubey H, Ranjan A. Recurrent liver metastasis in ovarian cancer. Ann Hepato-Biliary-Pancreat Surg. 2021;25(Suppl 1):S235. 10.14701/ahbps.EP-34.

[24] Clark AM, Ma B, Taylor DL, Griffith L, Wells A. Liver metastases: microenvironments and ex-vivo models. Exp Biol Med. 2016;241(15):1639–52. 10.1177/1535370216658144. 10.1177/1535370216658144 PMC4999624 27390264

[25] Li DF, Zhang X, Jiang LL. Molecular mechanism and potential therapeutic targets of liver metastasis from gastric cancer. Front Oncol. 2022;12:12. 10.3389/fonc.2022.1000807. 10.3389/fonc.2022.1000807 PMC9682021 36439439

[26] Gupta D, Lis CG. Pretreatment serum albumin as a predictor of cancer survival: a systematic review of the epidemiological literature. Nutr J. 2010;9:16. 10.1186/1475-2891-9-69. 21176210 10.1186/1475-2891-9-69 PMC3019132

[27] Issangya CE, Msuya D, Chilonga K, Herman A, Shao E, Shirima F, et al. Perioperative serum albumin as a predictor of adverse outcomes in abdominal surgery: prospective cohort hospital based study in Northern Tanzania. BMC Surg. 2020;20(1):7. 10.1186/s12893-020-00820-w. 32664910 10.1186/s12893-020-00820-w PMC7362485

[28] Muscaritoli M, Arends J, Bachmann P, Baracos V, Barthelemy N, Bertz H, Bozzetti F, Hutterer E, Isenring E, Kaasa S, Krznaric Z, Laird B, Larsson M, Laviano A, Muhlebach S, Oldervoll L, Ravasco P, Solheim TS, Strasser F, de van der Schueren M, Preiser JC, Bischoff SC. ESPEN practical guideline: clinical nutrition in cancer. Clin Nutr. 2021;40(5):2898–913. 10.1016/j.clnu.2021.02.005. PMID: WOS:000654716700009. 10.1016/j.clnu.2021.02.005 33946039

[29] Obling SR, Wilson BV, Pfeiffer P, Kjeldsen J. Home parenteral nutrition increases fat free mass in patients with incurable gastrointestinal cancer. Results of a randomized controlled trial. Clin Nutr. 2019;38(1):182–90. 10.1016/j.clnu.2017.12.011. 29305245 10.1016/j.clnu.2017.12.011

[30] Pascual-Anton L, Cardenes B, de la Cuesta RS, Gonzalez-Cortijo L, Lopez-Cabrera M, Cabanas C, et al. Mesothelial-to-mesenchymal transition and exosomes in peritoneal metastasis of ovarian cancer. Int J Mol Sci. 2021;22(21):17. 10.3390/ijms222111496. 10.3390/ijms222111496 PMC8584135 34768926

[31] Ma R, Ji ZH, Zhang Y, Li Y. Fundamental pathological mechanisms underlying gastro-intestinal cancer peritoneal metastasis. Zhonghua wei chang wai ke za zhi = Chinese journal of gastrointestinal surgery. 2021;24(3):198–203. 10.3760/cma.j.cn.441530-20201101-00583. 34645161 10.3760/cma.j.cn.441530-20201101-00583

[32] Kirkpatrick AW, Roberts DJ, De Waele J, Jaeschke R, Malbrain M, De Keulenaer B, Duchesne J, Bjorck M, Leppaniemi A, Ejike JC, Sugrue M, Cheatham M, Ivatury R, Ball CG, Blaser AR, Regli A, Balogh ZJ, D’Amours S, Debergh D, Kaplan M, Kimball E, Olvera C. Pediat guidelines Sub-Comm W. Intra-abdominal hypertension and the abdominal compartment syndrome: updated consensus definitions and clinical practice guidelines from the world society of the abdominal compartment syndrome. Intensive Care Med. 2013;39(7):1190–206. 10.1007/s00134-013-2906-z]. [PMID: WOS:000320334100003. 23673399 10.1007/s00134-013-2906-z PMC3680657

[33] Bouveresse S, Piton G, Badet N, Besch G, Pili-Floury S, Delabrousse E. Abdominal compartment syndrome and intra-abdominal hypertension in critically ill patients: diagnostic value of computed tomography. Eur Radiol. 2019;29(7):3839–46. 10.1007/s00330-018-5994-x. 30737569 10.1007/s00330-018-5994-x

[34] Razak OA, Yang SY, Cho MS, Min BS, Han YD. Palliative surgery as a bridge to systemic treatment for malignant bowel obstruction due to peritoneal metastases: a retrospective, case-control study. Asian J Surg. 2023;46(1):160–5. 10.1016/j.asjsur.2022.02.028. 35260331 10.1016/j.asjsur.2022.02.028

